# Supplementary material for: Coherent cross-modal generation of synthetic biomedical data to advance multimodal precision medicine
Source: PLoS Comput Biol. 2026 Apr 16;22(4):e1013455. doi: 10.1371/journal.pcbi.1013455 (PMC13108872; doi:10.1371/journal.pcbi.1013455)
Supplement: S1 Appendix — (PDF) [file pcbi.1013455.s001.pdf]

S1 Appendix: Distribution Fidelity for each Modality

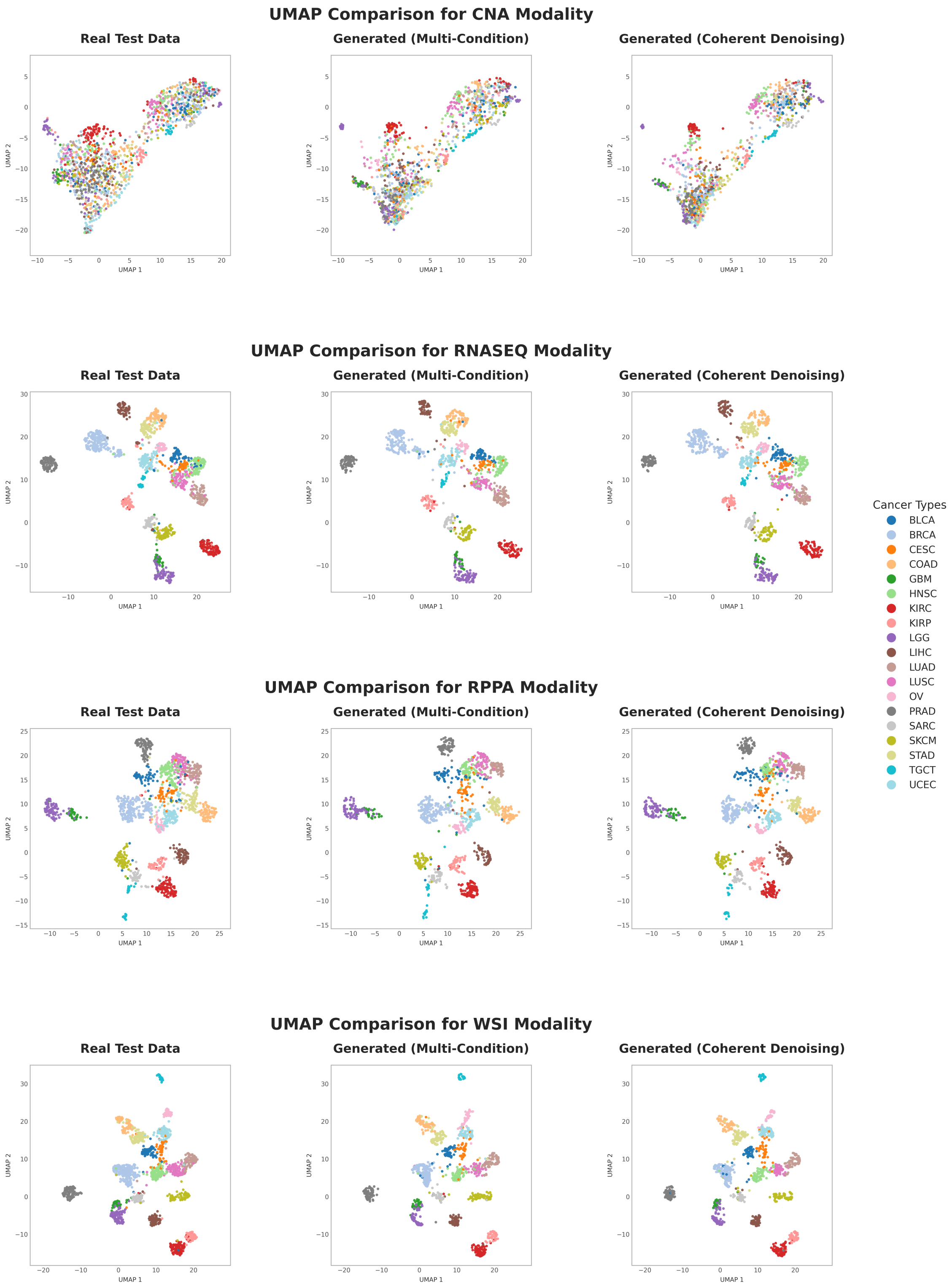

Figure A. Qualitative comparison of real and generated data manifolds across different data types with UMAP projections of the embeddings of each modality.
