# Supplementary material for: Coherent cross-modal generation of synthetic biomedical data to advance multimodal precision medicine
Source: PLoS Comput Biol. 2026 Apr 16;22(4):e1013455. doi: 10.1371/journal.pcbi.1013455 (PMC13108872; doi:10.1371/journal.pcbi.1013455)
Supplement: S2 Appendix — (PDF) [file pcbi.1013455.s002.pdf]

S2 Appendix: Full Reconstruction Accuracies

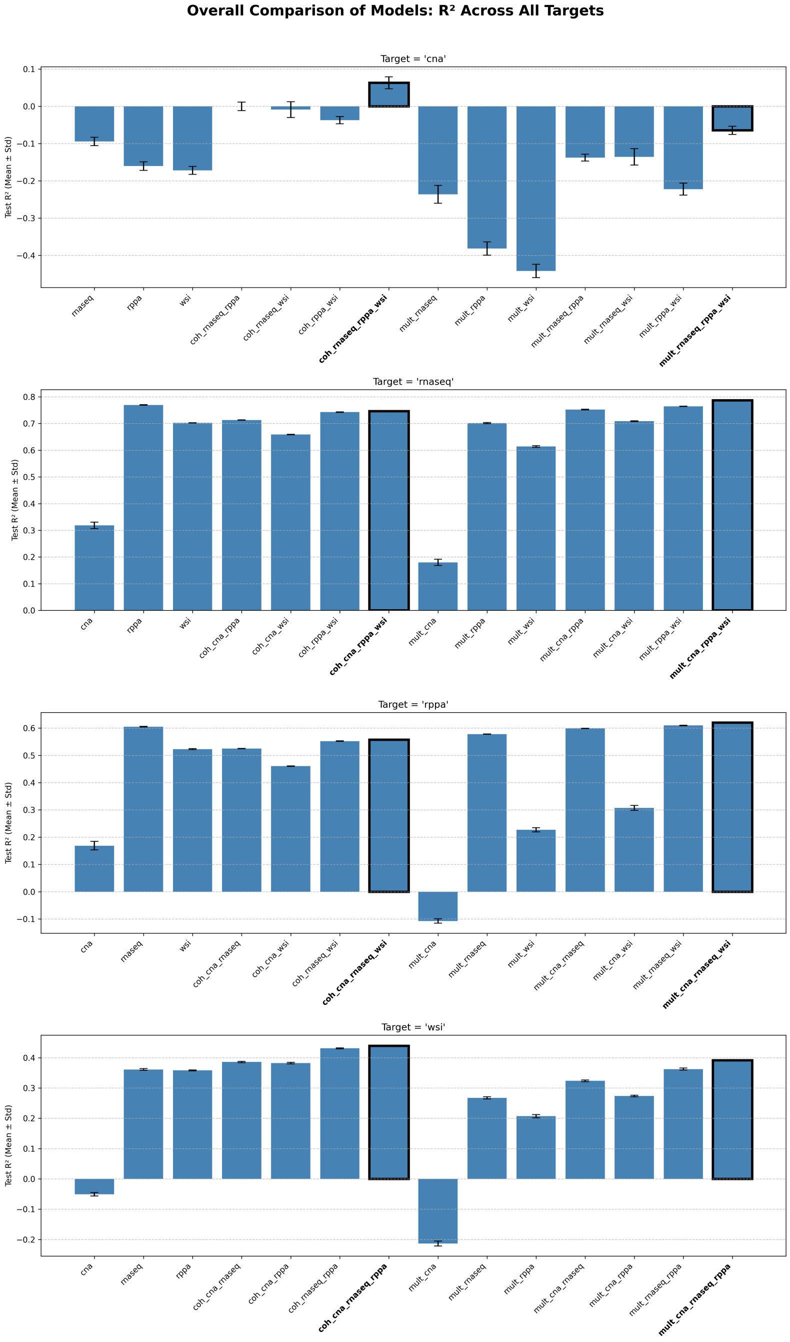

Figure A. Reconstruction accuracy (R^2) varies by target modality and generative model. Each panel displays the mean R^2 ($\pm$ standard deviation) from 10 generation runs for a different target modality. The x-axis compares the performance of single-condition models, the Coherent Denoising and multi-condition models, across different combinations of conditioning modalities. Highlighted bars indicate runs conditioned on all three other modalities.

Target modality	Source modalities	Single-condition	Coherent Denoising	Multi-condition
**CNA**	RPPA	-0.160 ± 0.012	—	-0.382 ± 0.018
	RNASEQ	-0.094 ± 0.011	—	-0.236 ± 0.024
	WSI	-0.172 ± 0.011	—	-0.442 ± 0.018
	RPPA + WSI	—	-0.037 ± 0.010	-0.222 ± 0.016
	RNASEQ + RPPA	—	-0.000 ± 0.011	-0.138 ± 0.009
	RNASEQ + WSI	—	-0.009 ± 0.021	-0.136 ± 0.022
	RNASEQ + RPPA + WSI	—	**0.063 ± 0.016**	-0.064 ± 0.011
**RNASEQ**	CNA	0.318 ± 0.012	—	0.180 ± 0.012
	RPPA	0.770 ± 0.001	—	0.702 ± 0.002
	WSI	0.702 ± 0.001	—	0.614 ± 0.003
	CNA + RPPA	—	0.713 ± 0.001	0.752 ± 0.001
	CNA + WSI	—	0.659 ± 0.001	0.709 ± 0.002
	RPPA + WSI	—	0.743 ± 0.001	0.765 ± 0.001
	CNA + RPPA + WSI	—	0.746 ± 0.001	**0.787 ± 0.001**
**RPPA**	CNA	0.169 ± 0.016	—	-0.107 ± 0.008
	RNASEQ	0.605 ± 0.001	—	0.578 ± 0.001
	WSI	0.523 ± 0.002	—	0.227 ± 0.008
	CNA + RNASEQ	—	0.525 ± 0.001	0.598 ± 0.001
	CNA + WSI	—	0.461 ± 0.001	0.307 ± 0.009
	RNASEQ + WSI	—	0.552 ± 0.001	0.610 ± 0.001
	CNA + RNASEQ + WSI	—	0.558 ± 0.001	**0.620 ± 0.001**
**WSI**	CNA	-0.051 ± 0.006	—	-0.213 ± 0.008
	RPPA	0.358 ± 0.001	—	0.207 ± 0.005
	RNASEQ	0.361 ± 0.003	—	0.267 ± 0.004
	CNA + RPPA	—	0.382 ± 0.003	0.274 ± 0.003
	CNA + RNASEQ	—	0.386 ± 0.002	0.324 ± 0.003
	RNASEQ + RPPA	—	0.431 ± 0.001	0.362 ± 0.003
	CNA + RNASEQ + RPPA	—	**0.439 ± 0.002**	0.392 ± 0.002

Table A. Expanded reconstruction accuracy (R^2) by target modality, source modalities, and generative method. Each block shows the mean R^2 (± standard deviation) from 10 independent generation runs for a different target modality. Rows indicate the source modality or combination of modalities used for conditioning. Columns indicate the generative method used. The best result for each target modality is highlighted in bold.

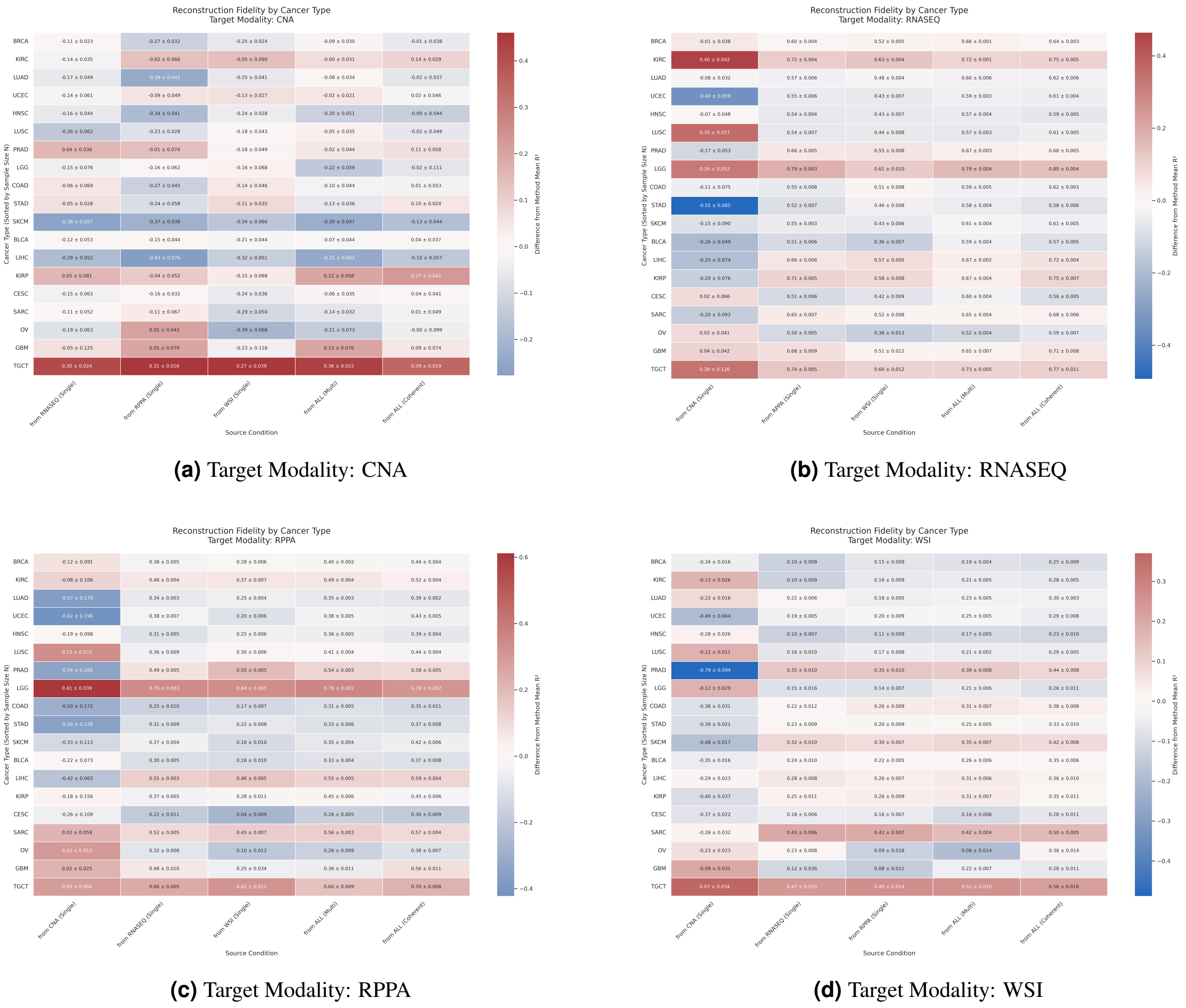

(a) Target Modality: CNA

(b) Target Modality: RNASEQ

(c) Target Modality: RPPA

(d) Target Modality: WSI

Figure B. **Stratified Reconstruction Fidelity (R^2) by Cancer Type and Target Modality.** Each panel corresponds to a different target modality: (a) CNA, (b) RNASEQ, (c) RPPA, and (d) WSI. Within each panel, rows represent the 20 individual cancer types (sorted by sample size), and columns represent the source condition used for generation. The "ALL (Multi)" and "ALL (Coherent)" columns represent generation using all three other modalities as input. Cell values report the mean $R^2 \pm$ standard deviation from 10 independent generation runs. The color scale indicates the performance on a cancer type relative to the mean performance across all cancer types for that specific method.
