# Supplementary material for: Coherent cross-modal generation of synthetic biomedical data to advance multimodal precision medicine
Source: PLoS Comput Biol. 2026 Apr 16;22(4):e1013455. doi: 10.1371/journal.pcbi.1013455 (PMC13108872; doi:10.1371/journal.pcbi.1013455)
Supplement: S3 Appendix — (PDF) [file pcbi.1013455.s003.pdf]

S3 Appendix: Preservation of Predictive Signals in Generated Data

Modality	Task	Data Source	Balanced Acc.	F1-Macro
**CNA**	Tumor Type	Real	0.565	0.563
		Synthetic (Coherent)	0.717	0.727
		Synthetic (Multi)	0.647	0.649
	Stage	Real	0.333	0.318
		Synthetic (Coherent)	0.379	0.369
		Synthetic (Multi)	0.363	0.352
**RNA-Seq**	Tumor Type	Real	0.929	0.928
		Synthetic (Coherent)	0.944	0.947
		Synthetic (Multi)	0.929	0.931
	Stage	Real	0.489	0.485
		Synthetic (Coherent)	0.498	0.490
		Synthetic (Multi)	0.510	0.504
**RPPA**	Tumor Type	Real	0.932	0.901
		Synthetic (Coherent)	0.933	0.935
		Synthetic (Multi)	0.918	0.919
	Stage	Real	0.481	0.488
		Synthetic (Coherent)	0.491	0.490
		Synthetic (Multi)	0.486	0.485
**WSI**	Tumor Type	Real	0.937	0.891
		Synthetic (Coherent)	0.938	0.939
		Synthetic (Multi)	0.904	0.906
	Stage	Real	0.505	0.501
		Synthetic (Coherent)	0.492	0.481
		Synthetic (Multi)	0.499	0.492

Table A. Performance metrics for Random Forest classifiers trained on single real modalities and tested on either real or synthetically generated data. Synthetic data performs on-par with real data.
