# Supplementary material for: Coherent cross-modal generation of synthetic biomedical data to advance multimodal precision medicine
Source: PLoS Comput Biol. 2026 Apr 16;22(4):e1013455. doi: 10.1371/journal.pcbi.1013455 (PMC13108872; doi:10.1371/journal.pcbi.1013455)
Supplement: S4 Appendix — (PDF) [file pcbi.1013455.s004.pdf]

S4 Appendix: Inference-Time Data Synthesis for Downstream Predictive Tasks

Data Condition	Experiment	Balanced Accuracy	Macro F1-Score
Full Data	Baseline	0.528±0.006	0.528±0.006
Cancer Label Only	Baseline	0.366±0.026	0.300±0.052
No CNA	Ablation	0.518±0.011	0.517±0.011
	Synthetic (Coherent)	0.530±0.007	0.528±0.008
	Synthetic (Multi)	0.527±0.008	0.526±0.009
No CNA, RNA-Seq	Ablation	0.468±0.014	0.463±0.015
	Synthetic (Coherent)	0.524±0.007	0.521±0.008
	Synthetic (Multi)	0.525±0.008	0.520±0.008
No CNA, RNA-Seq, RPPA	Ablation	0.398±0.009	0.326±0.022
	Synthetic (Coherent)	0.524±0.007	0.514±0.007
	Synthetic (Multi)	0.515±0.009	0.502±0.011
No CNA, RNA-Seq, WSI	Ablation	0.392±0.028	0.379±0.035
	Synthetic (Coherent)	0.518±0.008	0.515±0.008
	Synthetic (Multi)	0.499±0.009	0.496±0.009
No CNA, RPPA	Ablation	0.487±0.011	0.462±0.014
	Synthetic (Coherent)	0.533±0.008	0.528±0.008
	Synthetic (Multi)	0.526±0.006	0.520±0.007
No CNA, RPPA, WSI	Ablation	0.473±0.010	0.449±0.019
	Synthetic (Coherent)	0.519±0.005	0.516±0.005
	Synthetic (Multi)	0.520±0.005	0.518±0.005
No CNA, WSI	Ablation	0.497±0.005	0.493±0.005
	Synthetic (Coherent)	0.522±0.007	0.520±0.008
	Synthetic (Multi)	0.527±0.009	0.525±0.009
No RNA-Seq	Ablation	0.473±0.012	0.483±0.010
	Synthetic (Coherent)	0.523±0.004	0.520±0.004
	Synthetic (Multi)	0.531±0.008	0.526±0.008
No RNA-Seq, RPPA	Ablation	0.450±0.019	0.430±0.020
	Synthetic (Coherent)	0.521±0.006	0.513±0.007
	Synthetic (Multi)	0.523±0.005	0.514±0.005
No RNA-Seq, RPPA, WSI	Ablation	0.345±0.014	0.304±0.012
	Synthetic (Coherent)	0.429±0.011	0.427±0.011
	Synthetic (Multi)	0.421±0.007	0.417±0.007
No RNA-Seq, WSI	Ablation	0.349±0.018	0.321±0.026
	Synthetic (Coherent)	0.517±0.008	0.514±0.008
	Synthetic (Multi)	0.511±0.011	0.509±0.011
No RPPA	Ablation	0.508±0.007	0.491±0.009
	Synthetic (Coherent)	0.527±0.008	0.524±0.008
	Synthetic (Multi)	0.530±0.008	0.526±0.007
No RPPA, WSI	Ablation	0.473±0.009	0.462±0.008
	Synthetic (Coherent)	0.520±0.005	0.517±0.007
	Synthetic (Multi)	0.522±0.007	0.519±0.008
No WSI	Ablation	0.497±0.008	0.501±0.008
	Synthetic (Coherent)	0.522±0.009	0.521±0.009
	Synthetic (Multi)	0.523±0.010	0.522±0.011

Table A. Performance metrics for Random Forest classifier (mean and standard deviation across 10 repetitions) trained on the real multimodal training set and tested on the real data with simulated missing modalities, and synthetically generated data with the Coherent Denoisng method or with the multi-condition model.

Data Condition	Experiment Type	C-Index
Full Data	Baseline	0.736±0.003
Cancer Label Only	Baseline	0.536±0.048
No CNA	Ablation	0.711±0.007
	Synthetic (Coherent)	0.739±0.002
	Synthetic (Multi)	0.737±0.003
No CNA, RNA-Seq	Ablation	0.549±0.018
	Synthetic (Coherent)	0.735±0.003
	Synthetic (Multi)	0.733±0.003
No CNA, RNA-Seq, RPPA	Ablation	0.619±0.021
	Synthetic (Coherent)	0.726±0.003
	Synthetic (Multi)	0.714±0.004
No CNA, RNA-Seq, WSI	Ablation	0.517±0.017
	Synthetic (Coherent)	0.724±0.002
	Synthetic (Multi)	0.708±0.006
No CNA, RPPA	Ablation	0.701±0.005
	Synthetic (Coherent)	0.741±0.002
	Synthetic (Multi)	0.738±0.003
No CNA, RPPA, WSI	Ablation	0.636±0.018
	Synthetic (Coherent)	0.733±0.003
	Synthetic (Multi)	0.729±0.003
No CNA, WSI	Ablation	0.654±0.009
	Synthetic (Coherent)	0.729±0.002
	Synthetic (Multi)	0.728±0.003
No RNA-Seq	Ablation	0.574±0.016
	Synthetic (Coherent)	0.734±0.004
	Synthetic (Multi)	0.736±0.004
No RNA-Seq, RPPA	Ablation	0.622±0.012
	Synthetic (Coherent)	0.730±0.004
	Synthetic (Multi)	0.729±0.004
No RNA-Seq, RPPA, WSI	Ablation	0.563±0.021
	Synthetic (Coherent)	0.664±0.006
	Synthetic (Multi)	0.652±0.006
No RNA-Seq, WSI	Ablation	0.539±0.016
	Synthetic (Coherent)	0.725±0.004
	Synthetic (Multi)	0.718±0.004
No RPPA	Ablation	0.715±0.003
	Synthetic (Coherent)	0.738±0.004
	Synthetic (Multi)	0.735±0.004
No RPPA, WSI	Ablation	0.652±0.006
	Synthetic (Coherent)	0.728±0.004
	Synthetic (Multi)	0.724±0.003
No WSI	Ablation	0.689±0.003
	Synthetic (Coherent)	0.726±0.004
	Synthetic (Multi)	0.723±0.004

Table B. C-Index for Random Survival Forest (mean and standard deviation across 10 repetitions) trained on the real multimodal training set and tested on the real data with simulated missing modalities, and synthetically generated data with the Coherent Denoisng method or with the multi-condition model.
