# Supplementary material for: Coherent cross-modal generation of synthetic biomedical data to advance multimodal precision medicine
Source: PLoS Comput Biol. 2026 Apr 16;22(4):e1013455. doi: 10.1371/journal.pcbi.1013455 (PMC13108872; doi:10.1371/journal.pcbi.1013455)
Supplement: S5 Appendix — (PDF) [file pcbi.1013455.s005.pdf]

S5 Appendix: Counterfactual Analysis for Survival Analysis

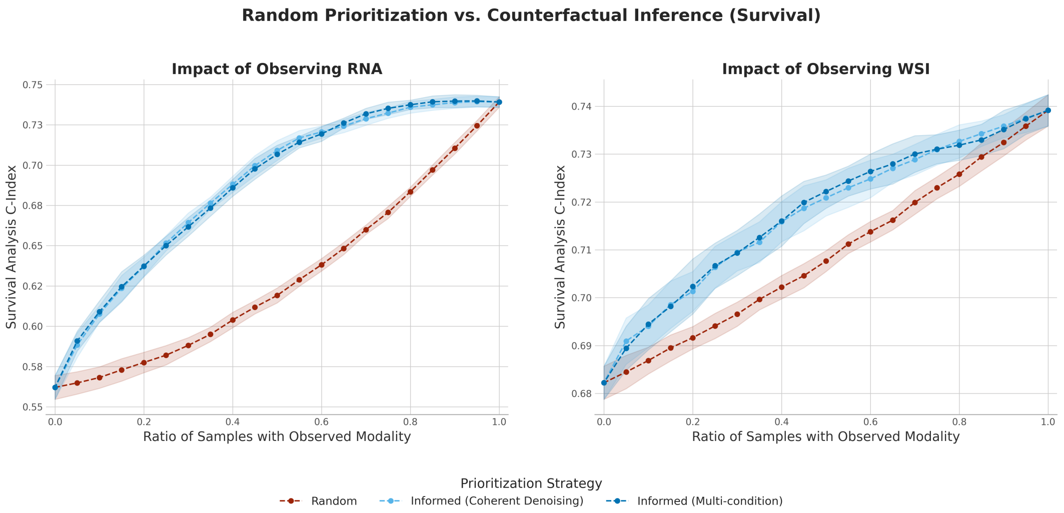

Figure A. Evaluating Counterfactual Inference for Prioritizing RNA-Seq (Left) and WSI (Right) Data Acquisition. The plot shows the Concordance Index of a multimodal random survival forest as the ratio of patients with an observed modality (RNA-Seq or WSI data) is varied. The Random Prioritization strategy (red) removes that modality data from patients at random. The Informed Prioritization strategies (blue) use a counterfactual variance score to preferentially acquire that modality data for the most informative patients first. Error bands show the standard deviation across 10 experimental repetitions. Note that the two plots are on separate y-axis scales, because of the intrinsic difference in performance that a predictive model has with and without that modality.
