# Supplementary material for: Coherent cross-modal generation of synthetic biomedical data to advance multimodal precision medicine
Source: PLoS Comput Biol. 2026 Apr 16;22(4):e1013455. doi: 10.1371/journal.pcbi.1013455 (PMC13108872; doi:10.1371/journal.pcbi.1013455)
Supplement: S6 Appendix — (PDF) [file pcbi.1013455.s006.pdf]

S6 Appendix: Statistical Tests

1 Preservation of Predictive Signals in Generated Data

To assess the statistical significance of performance differences between models using real vs. synthetic data, we employed a Repeated Measures ANOVA, with data modality and data source (real, coherent-generated, multi-generated) as within-subjects factors. Post-hoc analysis was conducted using paired t-tests, with p-values corrected for multiple comparisons using the Bonferroni method. A corrected alpha level was used to determine significance.

```
================================================================================
Repeated Measures ANOVA for:
  Task   : tumor_type
  Metric : balanced_accuracy
================================================================================
                      Anova
===================================================
                   F Value   Num DF  Den DF Pr > F
---------------------------------------------------
modality          20256.9274 3.0000 27.0000 0.0000
data_type           736.4593 2.0000 18.0000 0.0000
modality:data_type  346.9958 6.0000 54.0000 0.0000
===================================================



================================================================================
Performing Repeated Measures ANOVA for:
  Task   : tumor_type
  Metric : f1_macro
================================================================================
                     Anova
==================================================
                   F Value  Num DF  Den DF Pr > F
--------------------------------------------------
modality          6669.7201 3.0000 27.0000 0.0000
data_type          595.5192 2.0000 18.0000 0.0000
modality:data_type 126.5469 6.0000 54.0000 0.0000
==================================================



================================================================================
Performing Repeated Measures ANOVA for:
  Task   : stage
  Metric : balanced_accuracy
================================================================================
                     Anova
==================================================
                   F Value  Num DF  Den DF Pr > F
--------------------------------------------------
modality          1731.9448 3.0000 27.0000 0.0000
data_type           35.8453 2.0000 18.0000 0.0000
modality:data_type  20.0905 6.0000 54.0000 0.0000
==================================================



================================================================================
Performing Repeated Measures ANOVA for:
  Task   : stage
  Metric : f1_macro
================================================================================
                     Anova
==================================================
                   F Value  Num DF  Den DF Pr > F
--------------------------------------------------
modality          1367.5405 3.0000 27.0000 0.0000
data_type           13.8445 2.0000 18.0000 0.0002
modality:data_type  24.2893 6.0000 54.0000 0.0000
==================================================


================================================================================
T-tests for the effect of data_type
================================================================================
ANALYSIS: Task = TUMOR_TYPE, Metric = BALANCED_ACCURACY
================================================================================

--- Modality: cna ---
                     real vs. synthetic_from_coherent  : p-value = 0.000000 (Significant)
                     real vs. synthetic_from_multi     : p-value = 0.000000 (Significant)
```

```
  synthetic_from_coherent vs. synthetic_from_multi     : p-value = 0.000001 (Significant)

--- Modality: rnaseq ---
                     real vs. synthetic_from_coherent  : p-value = 0.000000 (Significant)
                     real vs. synthetic_from_multi     : p-value = 0.744300 (Not Significant)
  synthetic_from_coherent vs. synthetic_from_multi     : p-value = 0.000004 (Significant)

--- Modality: rppa ---
                     real vs. synthetic_from_coherent  : p-value = 0.357651 (Not Significant)
                     real vs. synthetic_from_multi     : p-value = 0.000001 (Significant)
  synthetic_from_coherent vs. synthetic_from_multi     : p-value = 0.000000 (Significant)

--- Modality: wsi ---
                     real vs. synthetic_from_coherent  : p-value = 0.506788 (Not Significant)
                     real vs. synthetic_from_multi     : p-value = 0.000000 (Significant)
  synthetic_from_coherent vs. synthetic_from_multi     : p-value = 0.000000 (Significant)
================================================================================
ANALYSIS: Task = TUMOR_TYPE, Metric = F1_MACRO
================================================================================

--- Modality: cna ---
                     real vs. synthetic_from_coherent  : p-value = 0.000000 (Significant)
                     real vs. synthetic_from_multi     : p-value = 0.000000 (Significant)
  synthetic_from_coherent vs. synthetic_from_multi     : p-value = 0.000001 (Significant)

--- Modality: rnaseq ---
                     real vs. synthetic_from_coherent  : p-value = 0.000000 (Significant)
                     real vs. synthetic_from_multi     : p-value = 0.017115 (Not Significant)
  synthetic_from_coherent vs. synthetic_from_multi     : p-value = 0.000000 (Significant)

--- Modality: rppa ---
                     real vs. synthetic_from_coherent  : p-value = 0.001034 (Significant)
                     real vs. synthetic_from_multi     : p-value = 0.034369 (Not Significant)
  synthetic_from_coherent vs. synthetic_from_multi     : p-value = 0.000000 (Significant)

--- Modality: wsi ---
                     real vs. synthetic_from_coherent  : p-value = 0.000000 (Significant)
                     real vs. synthetic_from_multi     : p-value = 0.000001 (Significant)
  synthetic_from_coherent vs. synthetic_from_multi     : p-value = 0.000000 (Significant)
================================================================================
ANALYSIS: Task = STAGE, Metric = BALANCED_ACCURACY
================================================================================

--- Modality: cna ---
                     real vs. synthetic_from_coherent  : p-value = 0.000019 (Significant)
                     real vs. synthetic_from_multi     : p-value = 0.000083 (Significant)
  synthetic_from_coherent vs. synthetic_from_multi     : p-value = 0.021267 (Not Significant)

--- Modality: rnaseq ---
                     real vs. synthetic_from_coherent  : p-value = 0.039307 (Not Significant)
                     real vs. synthetic_from_multi     : p-value = 0.000082 (Significant)
  synthetic_from_coherent vs. synthetic_from_multi     : p-value = 0.020446 (Not Significant)

--- Modality: rppa ---
                     real vs. synthetic_from_coherent  : p-value = 0.002267 (Significant)
                     real vs. synthetic_from_multi     : p-value = 0.246810 (Not Significant)
  synthetic_from_coherent vs. synthetic_from_multi     : p-value = 0.079922 (Not Significant)

--- Modality: wsi ---
                     real vs. synthetic_from_coherent  : p-value = 0.006409 (Significant)
                     real vs. synthetic_from_multi     : p-value = 0.083884 (Not Significant)
  synthetic_from_coherent vs. synthetic_from_multi     : p-value = 0.014765 (Significant)
================================================================================
ANALYSIS: Task = STAGE, Metric = F1_MACRO
================================================================================

--- Modality: cna ---
                     real vs. synthetic_from_coherent  : p-value = 0.000061 (Significant)
                     real vs. synthetic_from_multi     : p-value = 0.000143 (Significant)
  synthetic_from_coherent vs. synthetic_from_multi     : p-value = 0.033003 (Not Significant)

--- Modality: rnaseq ---
                     real vs. synthetic_from_coherent  : p-value = 0.317641 (Not Significant)
                     real vs. synthetic_from_multi     : p-value = 0.000516 (Significant)
  synthetic_from_coherent vs. synthetic_from_multi     : p-value = 0.013981 (Significant)

--- Modality: rppa ---
                     real vs. synthetic_from_coherent  : p-value = 0.502125 (Not Significant)
                     real vs. synthetic_from_multi     : p-value = 0.502480 (Not Significant)
  synthetic_from_coherent vs. synthetic_from_multi     : p-value = 0.194758 (Not Significant)

--- Modality: wsi ---
                     real vs. synthetic_from_coherent  : p-value = 0.000575 (Significant)
```

```
                    real vs. synthetic_from_multi     : p-value = 0.034262 (Not Significant)
  synthetic_from_coherent vs. synthetic_from_multi     : p-value = 0.002183 (Significant)
```

2 Inference-Time Data Synthesis for Downstream Predictive Tasks

To assess the statistical significance of the results from our downstream experiments, we employed a Repeated Measures ANOVA, with test condition and data source (ablated, coherent-generated, multi-generated) as within-subjects factors. Post-hoc analysis was conducted using paired t-tests, with p-values corrected for multiple comparisons using the Bonferroni method. A corrected alpha level was used to determine significance.

For the counterfactual analysis, we compared the overall performance of the prioritization strategies by calculating the Area Under the F1-Score Curve (AUC) for each of the 10 experimental repetitions and then performing a paired t-test on these AUC values. A corrected alpha level was used to determine significance in all post-hoc tests.

2.1 Stage Classification

```
================================================================================
ANOVA Results for Metric: BALANCED_ACCURACY
================================================================================
                          Anova
==========================================================
                          F Value   Num DF  Den DF  Pr > F
----------------------------------------------------------
test_condition            280.8313 13.0000 117.0000 0.0000
test_type                2542.4871  2.0000  18.0000 0.0000
test_condition:test_type   73.1597 26.0000 234.0000 0.0000
==========================================================



================================================================================
Post-Hoc Paired t-test Results for Metric: BALANCED_ACCURACY
================================================================================
Using Bonferroni-corrected alpha = 0.0012 for significance.


--- Condition: no_cna ---
          ablation vs. imputed_coherent  : p-value = 0.001249 (Not Significant)
          ablation vs. imputed_multi     : p-value = 0.071247 (Not Significant)
  imputed_coherent vs. imputed_multi     : p-value = 0.325872 (Not Significant)

--- Condition: no_rnaseq ---
          ablation vs. imputed_coherent  : p-value = 0.000000 (Significant)
          ablation vs. imputed_multi     : p-value = 0.000000 (Significant)
  imputed_coherent vs. imputed_multi     : p-value = 0.032120 (Not Significant)

--- Condition: no_rppa ---
          ablation vs. imputed_coherent  : p-value = 0.000267 (Significant)
          ablation vs. imputed_multi     : p-value = 0.000145 (Significant)
  imputed_coherent vs. imputed_multi     : p-value = 0.093417 (Not Significant)

--- Condition: no_wsi ---
          ablation vs. imputed_coherent  : p-value = 0.000011 (Significant)
          ablation vs. imputed_multi     : p-value = 0.000041 (Significant)
  imputed_coherent vs. imputed_multi     : p-value = 0.623052 (Not Significant)

--- Condition: no_cna_rnaseq ---
          ablation vs. imputed_coherent  : p-value = 0.000007 (Significant)
          ablation vs. imputed_multi     : p-value = 0.000001 (Significant)
  imputed_coherent vs. imputed_multi     : p-value = 0.787868 (Not Significant)

--- Condition: no_cna_rppa ---
          ablation vs. imputed_coherent  : p-value = 0.000001 (Significant)
          ablation vs. imputed_multi     : p-value = 0.000003 (Significant)
  imputed_coherent vs. imputed_multi     : p-value = 0.000671 (Significant)

--- Condition: no_cna_wsi ---
          ablation vs. imputed_coherent  : p-value = 0.000013 (Significant)
          ablation vs. imputed_multi     : p-value = 0.000005 (Significant)
  imputed_coherent vs. imputed_multi     : p-value = 0.166844 (Not Significant)

--- Condition: no_rnaseq_rppa ---
          ablation vs. imputed_coherent  : p-value = 0.000000 (Significant)
          ablation vs. imputed_multi     : p-value = 0.000000 (Significant)
  imputed_coherent vs. imputed_multi     : p-value = 0.562512 (Not Significant)

--- Condition: no_rnaseq_wsi ---
          ablation vs. imputed_coherent  : p-value = 0.000000 (Significant)
          ablation vs. imputed_multi     : p-value = 0.000000 (Significant)
  imputed_coherent vs. imputed_multi     : p-value = 0.099060 (Not Significant)

--- Condition: no_rppa_wsi ---
          ablation vs. imputed_coherent  : p-value = 0.000001 (Significant)
          ablation vs. imputed_multi     : p-value = 0.000001 (Significant)
```

```
  imputed_coherent vs. imputed_multi     : p-value = 0.492237 (Not Significant)

--- Condition: no_cna_rnaseq_rppa ---
          ablation vs. imputed_coherent  : p-value = 0.000000 (Significant)
          ablation vs. imputed_multi     : p-value = 0.000000 (Significant)
  imputed_coherent vs. imputed_multi     : p-value = 0.025485 (Not Significant)

--- Condition: no_cna_rnaseq_wsi ---
          ablation vs. imputed_coherent  : p-value = 0.000000 (Significant)
          ablation vs. imputed_multi     : p-value = 0.000001 (Significant)
  imputed_coherent vs. imputed_multi     : p-value = 0.000025 (Significant)

--- Condition: no_cna_rppa_wsi ---
          ablation vs. imputed_coherent  : p-value = 0.000000 (Significant)
          ablation vs. imputed_multi     : p-value = 0.000001 (Significant)
  imputed_coherent vs. imputed_multi     : p-value = 0.655039 (Not Significant)

--- Condition: no_rnaseq_rppa_wsi ---
          ablation vs. imputed_coherent  : p-value = 0.000000 (Significant)
          ablation vs. imputed_multi     : p-value = 0.000001 (Significant)
  imputed_coherent vs. imputed_multi     : p-value = 0.047397 (Not Significant)


================================================================================
ANOVA Results for Metric: MACRO_F1_SCORE
================================================================================
                          Anova
==========================================================
                          F Value   Num DF  Den DF  Pr > F
----------------------------------------------------------
test_condition            271.6872 13.0000 117.0000 0.0000
test_type                2079.5522  2.0000  18.0000 0.0000
test_condition:test_type   84.8627 26.0000 234.0000 0.0000
==========================================================




================================================================================
Post-Hoc Paired t-test Results for Metric: MACRO_F1_SCORE
================================================================================
Using Bonferroni-corrected alpha = 0.0012 for significance.


--- Condition: no_cna ---
          ablation vs. imputed_coherent  : p-value = 0.004239 (Not Significant)
          ablation vs. imputed_multi     : p-value = 0.080544 (Not Significant)
  imputed_coherent vs. imputed_multi     : p-value = 0.478453 (Not Significant)

--- Condition: no_rnaseq ---
          ablation vs. imputed_coherent  : p-value = 0.000004 (Significant)
          ablation vs. imputed_multi     : p-value = 0.000000 (Significant)
  imputed_coherent vs. imputed_multi     : p-value = 0.058794 (Not Significant)

--- Condition: no_rppa ---
          ablation vs. imputed_coherent  : p-value = 0.000014 (Significant)
          ablation vs. imputed_multi     : p-value = 0.000007 (Significant)
  imputed_coherent vs. imputed_multi     : p-value = 0.195575 (Not Significant)

--- Condition: no_wsi ---
          ablation vs. imputed_coherent  : p-value = 0.000088 (Significant)
          ablation vs. imputed_multi     : p-value = 0.000509 (Significant)
  imputed_coherent vs. imputed_multi     : p-value = 0.733080 (Not Significant)

--- Condition: no_cna_rnaseq ---
          ablation vs. imputed_coherent  : p-value = 0.000013 (Significant)
          ablation vs. imputed_multi     : p-value = 0.000001 (Significant)
  imputed_coherent vs. imputed_multi     : p-value = 0.807337 (Not Significant)

--- Condition: no_cna_rppa ---
          ablation vs. imputed_coherent  : p-value = 0.000000 (Significant)
          ablation vs. imputed_multi     : p-value = 0.000000 (Significant)
  imputed_coherent vs. imputed_multi     : p-value = 0.001391 (Not Significant)

--- Condition: no_cna_wsi ---
          ablation vs. imputed_coherent  : p-value = 0.000045 (Significant)
          ablation vs. imputed_multi     : p-value = 0.000007 (Significant)
  imputed_coherent vs. imputed_multi     : p-value = 0.143245 (Not Significant)

--- Condition: no_rnaseq_rppa ---
          ablation vs. imputed_coherent  : p-value = 0.000000 (Significant)
          ablation vs. imputed_multi     : p-value = 0.000000 (Significant)
  imputed_coherent vs. imputed_multi     : p-value = 0.757921 (Not Significant)
```

```
--- Condition: no_rnaseq_wsi ---
          ablation vs. imputed_coherent  : p-value = 0.000000 (Significant)
          ablation vs. imputed_multi     : p-value = 0.000000 (Significant)
  imputed_coherent vs. imputed_multi     : p-value = 0.133177 (Not Significant)

--- Condition: no_rppa_wsi ---
          ablation vs. imputed_coherent  : p-value = 0.000000 (Significant)
          ablation vs. imputed_multi     : p-value = 0.000000 (Significant)
  imputed_coherent vs. imputed_multi     : p-value = 0.581919 (Not Significant)

--- Condition: no_cna_rnaseq_rppa ---
          ablation vs. imputed_coherent  : p-value = 0.000000 (Significant)
          ablation vs. imputed_multi     : p-value = 0.000000 (Significant)
  imputed_coherent vs. imputed_multi     : p-value = 0.012593 (Not Significant)

--- Condition: no_cna_rnaseq_wsi ---
          ablation vs. imputed_coherent  : p-value = 0.000001 (Significant)
          ablation vs. imputed_multi     : p-value = 0.000003 (Significant)
  imputed_coherent vs. imputed_multi     : p-value = 0.000021 (Significant)

--- Condition: no_cna_rppa_wsi ---
          ablation vs. imputed_coherent  : p-value = 0.000002 (Significant)
          ablation vs. imputed_multi     : p-value = 0.000002 (Significant)
  imputed_coherent vs. imputed_multi     : p-value = 0.557439 (Not Significant)

--- Condition: no_rnaseq_rppa_wsi ---
          ablation vs. imputed_coherent  : p-value = 0.000000 (Significant)
          ablation vs. imputed_multi     : p-value = 0.000000 (Significant)
  imputed_coherent vs. imputed_multi     : p-value = 0.024217 (Not Significant)

================================================================================
ANALYSIS: Imputation vs. Full Data for Metric: BALANCED_ACCURACY
================================================================================
Using Bonferroni-corrected alpha = 0.0018 for significance.


--- Comparing Condition 'no_cna' against Full Data ---
  imputed_coherent vs. full_data         : p-value = 0.619192 (Not Significant)
     imputed_multi vs. full_data         : p-value = 0.605773 (Not Significant)

--- Comparing Condition 'no_rnaseq' against Full Data ---
  imputed_coherent vs. full_data         : p-value = 0.016879 (Not Significant)
     imputed_multi vs. full_data         : p-value = 0.536730 (Not Significant)

--- Comparing Condition 'no_rppa' against Full Data ---
  imputed_coherent vs. full_data         : p-value = 0.525017 (Not Significant)
     imputed_multi vs. full_data         : p-value = 0.675987 (Not Significant)

--- Comparing Condition 'no_wsi' against Full Data ---
  imputed_coherent vs. full_data         : p-value = 0.032724 (Not Significant)
     imputed_multi vs. full_data         : p-value = 0.151735 (Not Significant)

--- Comparing Condition 'no_cna_rnaseq' against Full Data ---
  imputed_coherent vs. full_data         : p-value = 0.126656 (Not Significant)
     imputed_multi vs. full_data         : p-value = 0.396201 (Not Significant)

--- Comparing Condition 'no_cna_rppa' against Full Data ---
  imputed_coherent vs. full_data         : p-value = 0.183691 (Not Significant)
     imputed_multi vs. full_data         : p-value = 0.313760 (Not Significant)

--- Comparing Condition 'no_cna_wsi' against Full Data ---
  imputed_coherent vs. full_data         : p-value = 0.047027 (Not Significant)
     imputed_multi vs. full_data         : p-value = 0.565660 (Not Significant)

--- Comparing Condition 'no_rnaseq_rppa' against Full Data ---
  imputed_coherent vs. full_data         : p-value = 0.039394 (Not Significant)
     imputed_multi vs. full_data         : p-value = 0.079447 (Not Significant)

--- Comparing Condition 'no_rnaseq_wsi' against Full Data ---
  imputed_coherent vs. full_data         : p-value = 0.000271 (Significant (full_data is better))
     imputed_multi vs. full_data         : p-value = 0.000121 (Significant (full_data is better))

--- Comparing Condition 'no_rppa_wsi' against Full Data ---
  imputed_coherent vs. full_data         : p-value = 0.013246 (Not Significant)
     imputed_multi vs. full_data         : p-value = 0.006305 (Not Significant)

--- Comparing Condition 'no_cna_rnaseq_rppa' against Full Data ---
  imputed_coherent vs. full_data         : p-value = 0.137558 (Not Significant)
     imputed_multi vs. full_data         : p-value = 0.010216 (Not Significant)

--- Comparing Condition 'no_cna_rnaseq_wsi' against Full Data ---
  imputed_coherent vs. full_data         : p-value = 0.000504 (Significant (full_data is better))
     imputed_multi vs. full_data         : p-value = 0.000002 (Significant (full_data is better))
```

```
--- Comparing Condition 'no_cna_rppa_wsi' against Full Data ---
  imputed_coherent vs. full_data         : p-value = 0.000538 (Significant (full_data is better))
     imputed_multi vs. full_data         : p-value = 0.005403 (Not Significant)

--- Comparing Condition 'no_rnaseq_rppa_wsi' against Full Data ---
  imputed_coherent vs. full_data         : p-value = 0.000000 (Significant (full_data is better))
     imputed_multi vs. full_data         : p-value = 0.000000 (Significant (full_data is better))


================================================================================
ANALYSIS: Imputation vs. Full Data for Metric: MACRO_F1_SCORE
================================================================================
Using Bonferroni-corrected alpha = 0.0018 for significance.


--- Comparing Condition 'no_cna' against Full Data ---
  imputed_coherent vs. full_data         : p-value = 0.970809 (Not Significant)
     imputed_multi vs. full_data         : p-value = 0.515542 (Not Significant)

--- Comparing Condition 'no_rnaseq' against Full Data ---
  imputed_coherent vs. full_data         : p-value = 0.002692 (Not Significant)
     imputed_multi vs. full_data         : p-value = 0.656059 (Not Significant)

--- Comparing Condition 'no_rppa' against Full Data ---
  imputed_coherent vs. full_data         : p-value = 0.176310 (Not Significant)
     imputed_multi vs. full_data         : p-value = 0.555354 (Not Significant)

--- Comparing Condition 'no_wsi' against Full Data ---
  imputed_coherent vs. full_data         : p-value = 0.013130 (Not Significant)
     imputed_multi vs. full_data         : p-value = 0.122376 (Not Significant)

--- Comparing Condition 'no_cna_rnaseq' against Full Data ---
  imputed_coherent vs. full_data         : p-value = 0.040785 (Not Significant)
     imputed_multi vs. full_data         : p-value = 0.083705 (Not Significant)

--- Comparing Condition 'no_cna_rppa' against Full Data ---
  imputed_coherent vs. full_data         : p-value = 0.922261 (Not Significant)
     imputed_multi vs. full_data         : p-value = 0.016928 (Not Significant)

--- Comparing Condition 'no_cna_wsi' against Full Data ---
  imputed_coherent vs. full_data         : p-value = 0.021443 (Not Significant)
     imputed_multi vs. full_data         : p-value = 0.281746 (Not Significant)

--- Comparing Condition 'no_rnaseq_rppa' against Full Data ---
  imputed_coherent vs. full_data         : p-value = 0.000937 (Significant (full_data is better))
     imputed_multi vs. full_data         : p-value = 0.000780 (Significant (full_data is better))

--- Comparing Condition 'no_rnaseq_wsi' against Full Data ---
  imputed_coherent vs. full_data         : p-value = 0.000202 (Significant (full_data is better))
     imputed_multi vs. full_data         : p-value = 0.000098 (Significant (full_data is better))

--- Comparing Condition 'no_rppa_wsi' against Full Data ---
  imputed_coherent vs. full_data         : p-value = 0.007403 (Not Significant)
     imputed_multi vs. full_data         : p-value = 0.002582 (Not Significant)

--- Comparing Condition 'no_cna_rnaseq_rppa' against Full Data ---
  imputed_coherent vs. full_data         : p-value = 0.001814 (Not Significant)
     imputed_multi vs. full_data         : p-value = 0.000429 (Significant (full_data is better))

--- Comparing Condition 'no_cna_rnaseq_wsi' against Full Data ---
  imputed_coherent vs. full_data         : p-value = 0.000191 (Significant (full_data is better))
     imputed_multi vs. full_data         : p-value = 0.000000 (Significant (full_data is better))

--- Comparing Condition 'no_cna_rppa_wsi' against Full Data ---
  imputed_coherent vs. full_data         : p-value = 0.000559 (Significant (full_data is better))
     imputed_multi vs. full_data         : p-value = 0.001492 (Significant (full_data is better))

--- Comparing Condition 'no_rnaseq_rppa_wsi' against Full Data ---
  imputed_coherent vs. full_data         : p-value = 0.000000 (Significant (full_data is better))
     imputed_multi vs. full_data         : p-value = 0.000000 (Significant (full_data is better))
```

2.2 Survival Analysis

```
================================================================================
ANOVA Results for Metric: C_INDEX
================================================================================
                          Anova
==========================================================
                          F Value   Num DF  Den DF  Pr > F
----------------------------------------------------------
```

```
test_condition             722.5935 13.0000 117.0000 0.0000
test_type                 1968.5437  4.0000  36.0000 0.0000
test_condition:test_type  154.8086 52.0000 468.0000 0.0000
==========================================================



================================================================================
Post-Hoc Paired t-test Results for Metric: C_INDEX
================================================================================
Using Bonferroni-corrected alpha = 0.0012 for significance.


--- Condition: no_cna ---
          ablation vs. imputed_coherent  : p-value = 0.000000 (Significant)
          ablation vs. imputed_multi     : p-value = 0.000001 (Significant)
  imputed_coherent vs. imputed_multi     : p-value = 0.063451 (Not Significant)

--- Condition: no_rnaseq ---
          ablation vs. imputed_coherent  : p-value = 0.000000 (Significant)
          ablation vs. imputed_multi     : p-value = 0.000000 (Significant)
  imputed_coherent vs. imputed_multi     : p-value = 0.026032 (Not Significant)

--- Condition: no_rppa ---
          ablation vs. imputed_coherent  : p-value = 0.000000 (Significant)
          ablation vs. imputed_multi     : p-value = 0.000000 (Significant)
  imputed_coherent vs. imputed_multi     : p-value = 0.001847 (Not Significant)

--- Condition: no_wsi ---
          ablation vs. imputed_coherent  : p-value = 0.000000 (Significant)
          ablation vs. imputed_multi     : p-value = 0.000000 (Significant)
  imputed_coherent vs. imputed_multi     : p-value = 0.027150 (Not Significant)

--- Condition: no_cna_rnaseq ---
          ablation vs. imputed_coherent  : p-value = 0.000000 (Significant)
          ablation vs. imputed_multi     : p-value = 0.000000 (Significant)
  imputed_coherent vs. imputed_multi     : p-value = 0.163051 (Not Significant)

--- Condition: no_cna_rppa ---
          ablation vs. imputed_coherent  : p-value = 0.000000 (Significant)
          ablation vs. imputed_multi     : p-value = 0.000000 (Significant)
  imputed_coherent vs. imputed_multi     : p-value = 0.003526 (Not Significant)

--- Condition: no_cna_wsi ---
          ablation vs. imputed_coherent  : p-value = 0.000000 (Significant)
          ablation vs. imputed_multi     : p-value = 0.000000 (Significant)
  imputed_coherent vs. imputed_multi     : p-value = 0.530708 (Not Significant)

--- Condition: no_rnaseq_rppa ---
          ablation vs. imputed_coherent  : p-value = 0.000000 (Significant)
          ablation vs. imputed_multi     : p-value = 0.000000 (Significant)
  imputed_coherent vs. imputed_multi     : p-value = 0.095726 (Not Significant)

--- Condition: no_rnaseq_wsi ---
          ablation vs. imputed_coherent  : p-value = 0.000000 (Significant)
          ablation vs. imputed_multi     : p-value = 0.000000 (Significant)
  imputed_coherent vs. imputed_multi     : p-value = 0.001713 (Not Significant)

--- Condition: no_rppa_wsi ---
          ablation vs. imputed_coherent  : p-value = 0.000000 (Significant)
          ablation vs. imputed_multi     : p-value = 0.000000 (Significant)
  imputed_coherent vs. imputed_multi     : p-value = 0.005730 (Not Significant)

--- Condition: no_cna_rnaseq_rppa ---
          ablation vs. imputed_coherent  : p-value = 0.000000 (Significant)
          ablation vs. imputed_multi     : p-value = 0.000000 (Significant)
  imputed_coherent vs. imputed_multi     : p-value = 0.000060 (Significant)

--- Condition: no_cna_rnaseq_wsi ---
          ablation vs. imputed_coherent  : p-value = 0.000000 (Significant)
          ablation vs. imputed_multi     : p-value = 0.000000 (Significant)
  imputed_coherent vs. imputed_multi     : p-value = 0.000020 (Significant)

--- Condition: no_cna_rppa_wsi ---
          ablation vs. imputed_coherent  : p-value = 0.000000 (Significant)
          ablation vs. imputed_multi     : p-value = 0.000000 (Significant)
  imputed_coherent vs. imputed_multi     : p-value = 0.021486 (Not Significant)

--- Condition: no_rnaseq_rppa_wsi ---
          ablation vs. imputed_coherent  : p-value = 0.000000 (Significant)
          ablation vs. imputed_multi     : p-value = 0.000000 (Significant)
  imputed_coherent vs. imputed_multi     : p-value = 0.003909 (Not Significant)
```

```
================================================================================
ANALYSIS: Imputation vs. Full Data for Metric: C_INDEX
================================================================================
Using Bonferroni-corrected alpha = 0.0018 for significance.


--- Comparing Condition 'no_cna' against Full Data ---
  imputed_coherent vs. full_data         : p-value = 0.008620 (Not Significant)
     imputed_multi vs. full_data         : p-value = 0.322543 (Not Significant)

--- Comparing Condition 'no_rnaseq' against Full Data ---
  imputed_coherent vs. full_data         : p-value = 0.021880 (Not Significant)
     imputed_multi vs. full_data         : p-value = 0.885021 (Not Significant)

--- Comparing Condition 'no_rppa' against Full Data ---
  imputed_coherent vs. full_data         : p-value = 0.008366 (Not Significant)
     imputed_multi vs. full_data         : p-value = 0.100889 (Not Significant)

--- Comparing Condition 'no_wsi' against Full Data ---
  imputed_coherent vs. full_data         : p-value = 0.000000 (Significant (full_data is better))
     imputed_multi vs. full_data         : p-value = 0.000001 (Significant (full_data is better))

--- Comparing Condition 'no_cna_rnaseq' against Full Data ---
  imputed_coherent vs. full_data         : p-value = 0.497254 (Not Significant)
     imputed_multi vs. full_data         : p-value = 0.025909 (Not Significant)

--- Comparing Condition 'no_cna_rppa' against Full Data ---
  imputed_coherent vs. full_data         : p-value = 0.000585 (Significant (imputed_coherent is better))
     imputed_multi vs. full_data         : p-value = 0.221189 (Not Significant)

--- Comparing Condition 'no_cna_wsi' against Full Data ---
  imputed_coherent vs. full_data         : p-value = 0.001057 (Significant (full_data is better))
     imputed_multi vs. full_data         : p-value = 0.000103 (Significant (full_data is better))

--- Comparing Condition 'no_rnaseq_rppa' against Full Data ---
  imputed_coherent vs. full_data         : p-value = 0.000046 (Significant (full_data is better))
     imputed_multi vs. full_data         : p-value = 0.000014 (Significant (full_data is better))

--- Comparing Condition 'no_rnaseq_wsi' against Full Data ---
  imputed_coherent vs. full_data         : p-value = 0.000003 (Significant (full_data is better))
     imputed_multi vs. full_data         : p-value = 0.000001 (Significant (full_data is better))

--- Comparing Condition 'no_rppa_wsi' against Full Data ---
  imputed_coherent vs. full_data         : p-value = 0.000941 (Significant (full_data is better))
     imputed_multi vs. full_data         : p-value = 0.000001 (Significant (full_data is better))

--- Comparing Condition 'no_cna_rnaseq_rppa' against Full Data ---
  imputed_coherent vs. full_data         : p-value = 0.000037 (Significant (full_data is better))
     imputed_multi vs. full_data         : p-value = 0.000000 (Significant (full_data is better))

--- Comparing Condition 'no_cna_rnaseq_wsi' against Full Data ---
  imputed_coherent vs. full_data         : p-value = 0.000000 (Significant (full_data is better))
     imputed_multi vs. full_data         : p-value = 0.000000 (Significant (full_data is better))

--- Comparing Condition 'no_cna_rppa_wsi' against Full Data ---
  imputed_coherent vs. full_data         : p-value = 0.086749 (Not Significant)
     imputed_multi vs. full_data         : p-value = 0.001257 (Significant (full_data is better))

--- Comparing Condition 'no_rnaseq_rppa_wsi' against Full Data ---
  imputed_coherent vs. full_data         : p-value = 0.000000 (Significant (full_data is better))
     imputed_multi vs. full_data         : p-value = 0.000000 (Significant (full_data is better))
```

3 Counterfactual Analysis

```
================================================================================
Statistical Analysis: Counterfactual Inference vs. Random Ablation
Metric: Area Under the F1-Score Curve (AUC)
================================================================================
Using Bonferroni-corrected alpha = 0.0167 for significance.


--- Analysis for Ablated Modality: RNA ---
    random vs. coherent  : p-value = 0.000000 (Significant (coherent is better))
    random vs. multi     : p-value = 0.000000 (Significant (multi is better))
  coherent vs. multi     : p-value = 0.626752 (Not Significant)

--- Analysis for Ablated Modality: WSI ---
    random vs. coherent  : p-value = 0.000000 (Significant (coherent is better))
    random vs. multi     : p-value = 0.000000 (Significant (multi is better))
  coherent vs. multi     : p-value = 0.057699 (Not Significant)
```
