# Supplementary material for: Coherent cross-modal generation of synthetic biomedical data to advance multimodal precision medicine
Source: PLoS Comput Biol. 2026 Apr 16;22(4):e1013455. doi: 10.1371/journal.pcbi.1013455 (PMC13108872; doi:10.1371/journal.pcbi.1013455)
Supplement: S7 Appendix — (PDF) [file pcbi.1013455.s007.pdf]

S7 Appendix: Hyperparameters

Model (Target/Condition)	batch_size	learning_rate	initial_size	n_layers	time_embedding_dimension	cond_embedding_dim
cna_from_rnaseq	64	0.0001	1024	5	128	32
cna_from_rppa	128	0.0001	1024	5	128	32
cna_from_wsi	128	0.0001	1024	5	128	32
cna_from_multi	128	0.0001	1024	4	128	32
rnaseq_from_cna	128	0.0001	1024	5	64	32
rnaseq_from_rppa	128	0.0001	1024	5	64	32
rnaseq_from_wsi	64	0.0001	1024	7	64	32
rnaseq_from_multi	128	0.0001	1024	5	64	32
rppa_from_cna	128	0.0001	1024	6	64	32
rppa_from_rnaseq	128	0.0001	1024	7	128	32
rppa_from_wsi	128	0.0001	1024	6	64	32
rppa_from_multi	128	0.0001	1024	6	128	32
wsi_from_cna	128	0.0001	1024	6	64	32
wsi_from_rnaseq	128	0.0001	1024	5	64	32
wsi_from_rppa	128	0.0001	1024	6	128	32
wsi_from_multi	128	0.0001	1024	7	64	32

Table A. Best hyperparmeters for each trained model
