# Supplementary material for: Coherent cross-modal generation of synthetic biomedical data to advance multimodal precision medicine
Source: PLoS Comput Biol. 2026 Apr 16;22(4):e1013455. doi: 10.1371/journal.pcbi.1013455 (PMC13108872; doi:10.1371/journal.pcbi.1013455)
Supplement: S8 Appendix — (PDF) [file pcbi.1013455.s008.pdf]

S8 Appendix: Coherent Denoising Ensemble Weights

This appendix details the performance-based weighting scheme used in the Coherent Denoising ensemble. The weights are assigned to prioritize high-fidelity modalities while down-weighting noisier inputs, ensuring the generation process is driven by the most reliable available signal.

Target Modality	Source Modality	Validation Loss (MSE)
**CNA**	RNA-Seq	1.006
	RPPA	1.096
	WSI	1.086
**RNA-Seq**	CNA	0.640
	RPPA	0.220
	WSI	0.294
**RPPA**	CNA	0.778
	RNA-Seq	0.367
	WSI	0.437
**WSI**	CNA	1.049
	RNA-Seq	0.642
	RPPA	0.636

Table A. Validation Mean Squared Error (MSE) per Conditioning Pair. The weights (w_i) for the ensemble are calculated as the Softmax of the inverse validation MSE ($1/L$) for each available single-condition model. The table below lists the raw MSE values (L) used for this calculation. Lower MSE indicates higher reconstruction fidelity for that specific source-target pair.

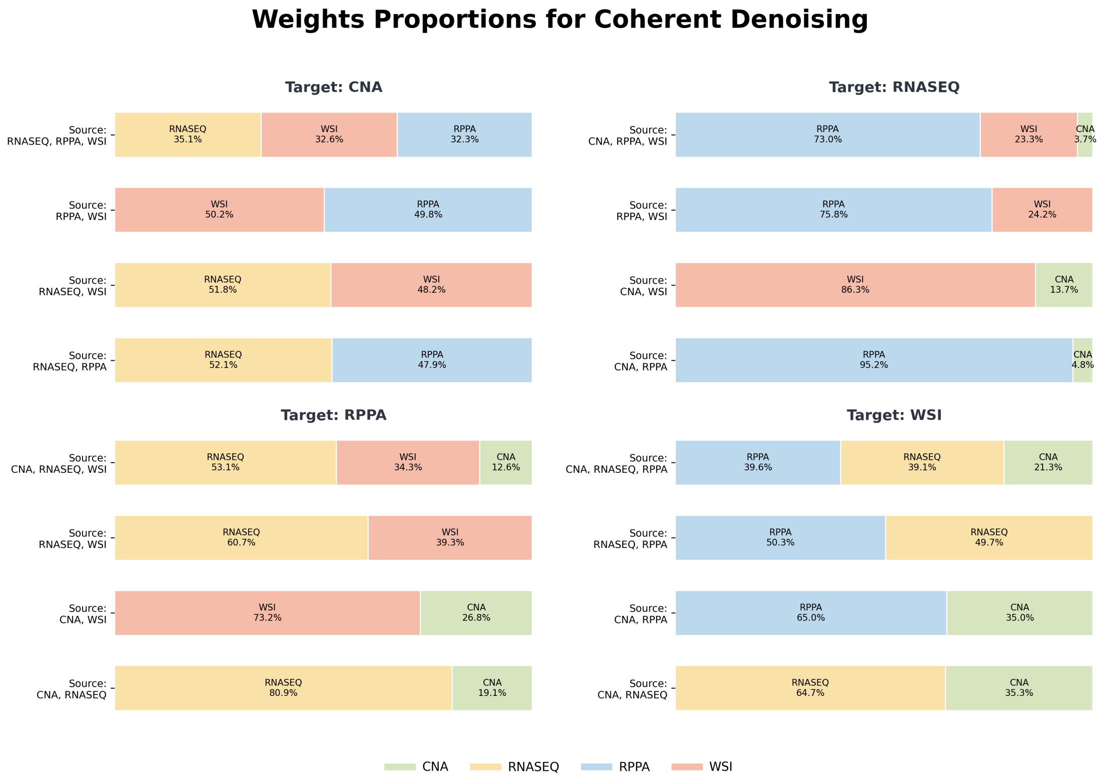

Figure A. Modality-Specific Influence in the Coherent Denoising Ensemble. Each panel illustrates the relative contribution (consensus share) of available conditioning modalities toward the generation of a specific target modality (CNA, RNA-Seq, RPPA, or WSI). Within each stacked bar, the segments are ordered from the highest influence to the lowest , reflecting the hierarchical prioritization of the models.
