# Supplementary material for: Coherent cross-modal generation of synthetic biomedical data to advance multimodal precision medicine
Source: PLoS Comput Biol. 2026 Apr 16;22(4):e1013455. doi: 10.1371/journal.pcbi.1013455 (PMC13108872; doi:10.1371/journal.pcbi.1013455)
Supplement: S9 Appendix — (PDF) [file pcbi.1013455.s009.pdf]

S9 Appendix: Sensitivity Analysis of Rejection Sampling

This appendix presents a sensitivity analysis of the Coherent Denoising rejection sampling mechanism. To evaluate the robustness of the framework, we show the effect of varying the cosine distance threshold (τ_{cos}) across a range of ±30% from the default value of 1.0.

**Cosine Threshold**	**cna**	**rnaseq**	**rppa**	**wsi**
**0.7 (-30%)**	21.81% ± 0.69	7.74% ± 0.79	16.38% ± 0.57	13.22% ± 0.42
**0.8 (-20%)**	8.61% ± 0.59	2.69% ± 0.37	5.76% ± 0.37	4.07% ± 0.56
**0.9 (-10%)**	2.44% ± 0.47	0.93% ± 0.17	1.76% ± 0.31	1.10% ± 0.16
**1.0 (default)**	0.32% ± 0.16	0.24% ± 0.11	0.44% ± 0.14	0.26% ± 0.08
**1.1 (+10%)**	0.02% ± 0.03	0.07% ± 0.07	0.03% ± 0.04	0.05% ± 0.06
**1.2 (+20%)**	0.00% ± 0.00	0.00% ± 0.00	0.01% ± 0.02	0.00% ± 0.00
**1.3 (+30%)**	0.00% ± 0.00	0.00% ± 0.00	0.00% ± 0.00	0.00% ± 0.00

Table A. Impact of Cosine Threshold on Rejection Rate. The table reports the mean Rejection Rate ± standard deviation across 10 independent experimental runs.

**Cosine Threshold**	**cna**	**rnaseq**	**rppa**	**wsi**
**0.7 (-30%)**	0.0422 ± 0.0183	0.7975 ± 0.0005	0.6433 ± 0.0010	0.4524 ± 0.0015
**0.8 (-20%)**	0.0518 ± 0.0128	0.7978 ± 0.0007	0.6441 ± 0.0009	0.4520 ± 0.0014
**0.9 (-10%)**	0.0453 ± 0.0126	0.7978 ± 0.0006	0.6439 ± 0.0011	0.4526 ± 0.0008
**1.0 (default)**	0.0407 ± 0.0052	0.7980 ± 0.0004	0.6443 ± 0.0013	0.4524 ± 0.0019
**1.1 (+10%)**	0.0464 ± 0.0090	0.7977 ± 0.0007	0.6442 ± 0.0009	0.4527 ± 0.0017
**1.2 (+20%)**	0.0516 ± 0.0153	0.7978 ± 0.0005	0.6444 ± 0.0012	0.4530 ± 0.0017
**1.3 (+30%)**	0.0469 ± 0.0147	0.7978 ± 0.0004	0.6437 ± 0.0011	0.4520 ± 0.0021

Table B. Impact of Cosine Threshold on Reconstruction Fidelity. The table reports the mean Reconstruction Error (R^2) ± standard deviation across 10 independent experimental runs. To provide an estimate of stability, R^2 scores were calculated on the full generated cohort, including samples that would have been rejected at that specific threshold.
