# Supplementary material for: Coherent cross-modal generation of synthetic biomedical data to advance multimodal precision medicine
Source: PLoS Comput Biol. 2026 Apr 16;22(4):e1013455. doi: 10.1371/journal.pcbi.1013455 (PMC13108872; doi:10.1371/journal.pcbi.1013455)
Supplement: S10 Appendix — (PDF) [file pcbi.1013455.s010.pdf]

S10 Appendix: Impact of Embedding Dimension

In this appendix we compare the default latent dimension (32) against larger dimensions (64, 128, 256). This analysis evaluates both the baseline predictive performance of the real data and the generative capability of the framework in higher-dimensional spaces.

UMAP

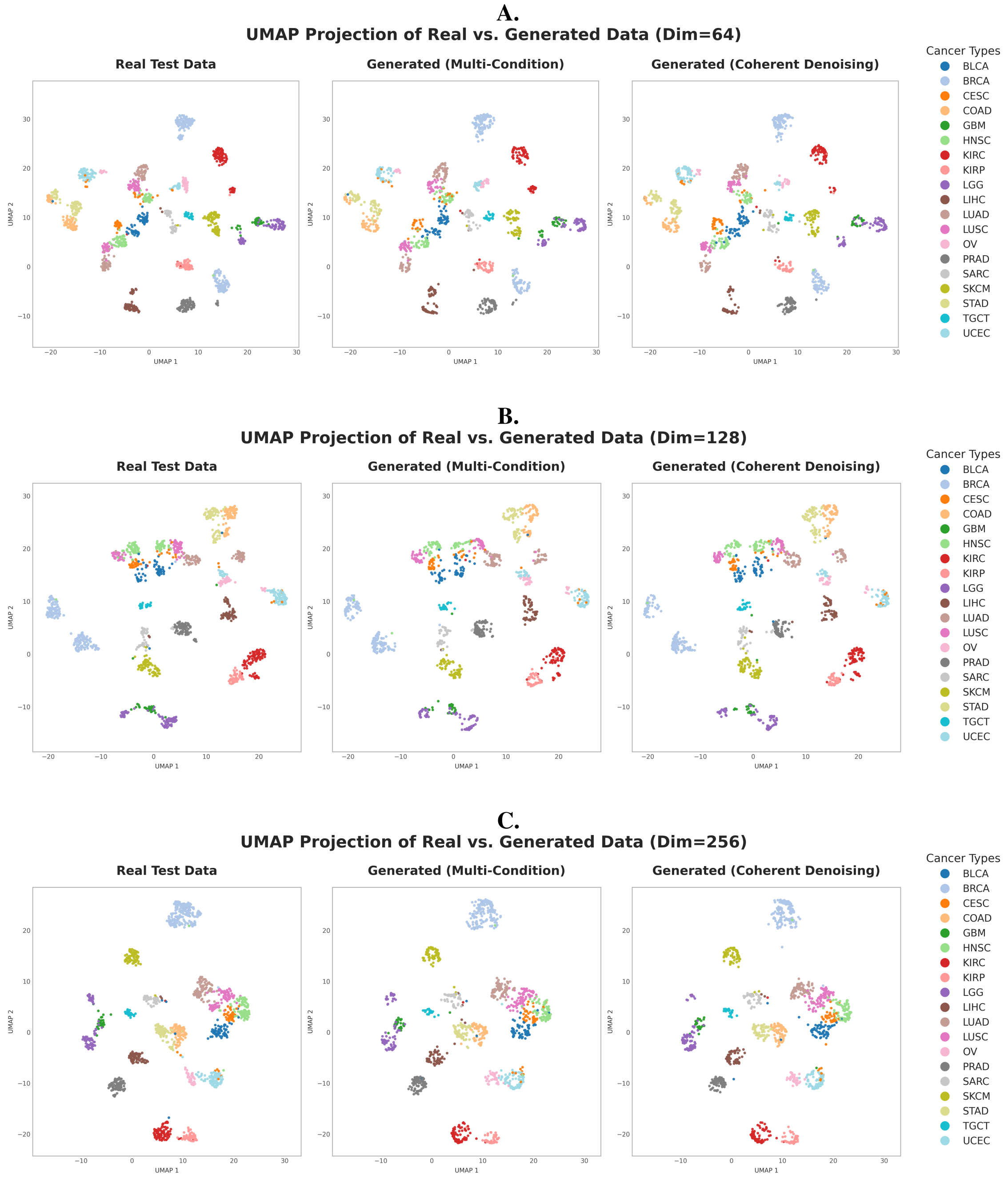

Figure A. UMAP projections across Latent Dimensions. UMAP projections of multimodal embeddings for dimensions 64 (A), 128 (B), and 256 (C). In each row, the ground-truth data (Left) is compared with reconstructions from the Multi-condition model (Middle) and the Coherent Denoising ensemble (Right). The visualization confirms that the generative framework maintains structural integrity and distinct cancer-type clustering even as the dimensionality of the latent space increases.

Downstream Tasks

Embedding Dim	Stage Classification (F1 Score)	Survival Analysis (C-Index)
**32 (Default)**	**0.528 ± 0.006**	**0.736 ± 0.003**
**64**	0.519 ± 0.008	0.732 ± 0.004
**128**	0.511 ± 0.008	0.734 ± 0.004
**256**	0.513 ± 0.014	0.727 ± 0.003

Table A. Baseline Performance of Real Data across Latent Dimensions. We evaluated the predictive performance of Multimodal Random Forest classifiers and Random Survival Forests trained on the real data, and tested on ground-truth embeddings at varying dimensions. Values represent Mean ± Standard Deviation across 10 experimental runs.

Embedding Dim	Test Condition	Stage Classification (F1 Score)			Survival Analysis (C-Index)		
		Ablation	Gain from generated data		Ablation	Gain from generated data	
		Drop	Multi-cond.	Coherent	Drop	Multi-cond.	Coherent
**64**	No Cna	-0.011 ± 0.014	+0.007 ± 0.015	+0.012 ± 0.014	-0.030 ± 0.005	+0.032 ± 0.006	+0.031 ± 0.006
	No Rnaseq	-0.056 ± 0.023	+0.058 ± 0.017	+0.055 ± 0.018	-0.166 ± 0.006	+0.167 ± 0.008	+0.167 ± 0.008
	No Rppa	-0.046 ± 0.010	+0.046 ± 0.016	+0.050 ± 0.014	-0.024 ± 0.004	+0.028 ± 0.003	+0.026 ± 0.003
	No Wsi	-0.030 ± 0.011	+0.031 ± 0.011	+0.031 ± 0.013	-0.048 ± 0.005	+0.038 ± 0.005	+0.044 ± 0.006
	No Cna, Rnaseq	-0.062 ± 0.024	+0.050 ± 0.023	+0.062 ± 0.021	-0.179 ± 0.016	+0.177 ± 0.017	+0.182 ± 0.015
	No Cna, Rppa	-0.067 ± 0.013	+0.065 ± 0.011	+0.068 ± 0.013	-0.048 ± 0.009	+0.051 ± 0.010	+0.050 ± 0.011
	No Cna, Wsi	-0.033 ± 0.010	+0.035 ± 0.014	+0.031 ± 0.014	-0.080 ± 0.008	+0.073 ± 0.009	+0.078 ± 0.008
	No Rnaseq, Rppa	-0.085 ± 0.029	+0.068 ± 0.026	+0.073 ± 0.029	-0.147 ± 0.008	+0.146 ± 0.010	+0.146 ± 0.009
	No Rnaseq, Wsi	-0.243 ± 0.032	+0.232 ± 0.034	+0.237 ± 0.035	-0.172 ± 0.013	+0.162 ± 0.014	+0.166 ± 0.013
	No Rppa, Wsi	-0.065 ± 0.008	+0.065 ± 0.008	+0.062 ± 0.009	-0.087 ± 0.007	+0.079 ± 0.007	+0.083 ± 0.007
	No Cna, Rnaseq, Rppa	-0.156 ± 0.035	+0.136 ± 0.034	+0.147 ± 0.038	-0.163 ± 0.007	+0.158 ± 0.007	+0.162 ± 0.009
	No Cna, Rnaseq, Wsi	-0.229 ± 0.039	+0.200 ± 0.044	+0.225 ± 0.039	-0.184 ± 0.015	+0.168 ± 0.011	+0.180 ± 0.013
	No Cna, Rppa, Wsi	-0.081 ± 0.015	+0.081 ± 0.015	+0.076 ± 0.016	-0.114 ± 0.015	+0.106 ± 0.015	+0.112 ± 0.015
	No Rnaseq, Rppa, Wsi	-0.260 ± 0.035	+0.144 ± 0.034	+0.160 ± 0.030	-0.168 ± 0.018	+0.094 ± 0.024	+0.111 ± 0.021
**128**	No Cna	-0.017 ± 0.011	+0.022 ± 0.010	+0.020 ± 0.010	-0.039 ± 0.010	+0.038 ± 0.009	+0.037 ± 0.010
	No Rnaseq	-0.084 ± 0.019	+0.090 ± 0.020	+0.089 ± 0.022	-0.184 ± 0.006	+0.182 ± 0.008	+0.185 ± 0.007
	No Rppa	-0.037 ± 0.011	+0.042 ± 0.013	+0.039 ± 0.012	-0.024 ± 0.007	+0.027 ± 0.006	+0.026 ± 0.006
	No Wsi	-0.027 ± 0.010	+0.031 ± 0.009	+0.033 ± 0.008	-0.038 ± 0.010	+0.029 ± 0.007	+0.031 ± 0.008
	No Cna, Rnaseq	-0.094 ± 0.035	+0.094 ± 0.030	+0.099 ± 0.032	-0.197 ± 0.013	+0.189 ± 0.011	+0.196 ± 0.012
	No Cna, Rppa	-0.066 ± 0.008	+0.061 ± 0.011	+0.064 ± 0.010	-0.055 ± 0.011	+0.059 ± 0.012	+0.056 ± 0.010
	No Cna, Wsi	-0.042 ± 0.011	+0.048 ± 0.009	+0.046 ± 0.010	-0.091 ± 0.008	+0.082 ± 0.008	+0.081 ± 0.007
	No Rnaseq, Rppa	-0.080 ± 0.028	+0.080 ± 0.028	+0.078 ± 0.027	-0.166 ± 0.010	+0.162 ± 0.009	+0.163 ± 0.011
	No Rnaseq, Wsi	-0.279 ± 0.035	+0.270 ± 0.031	+0.284 ± 0.037	-0.149 ± 0.010	+0.136 ± 0.008	+0.145 ± 0.007
	No Rppa, Wsi	-0.047 ± 0.009	+0.050 ± 0.010	+0.056 ± 0.007	-0.089 ± 0.008	+0.083 ± 0.007	+0.085 ± 0.008
	No Cna, Rnaseq, Rppa	-0.125 ± 0.032	+0.116 ± 0.033	+0.116 ± 0.028	-0.196 ± 0.019	+0.181 ± 0.018	+0.187 ± 0.018
	No Cna, Rnaseq, Wsi	-0.240 ± 0.041	+0.221 ± 0.034	+0.242 ± 0.037	-0.176 ± 0.017	+0.152 ± 0.015	+0.169 ± 0.013
	No Cna, Rppa, Wsi	-0.085 ± 0.016	+0.093 ± 0.016	+0.091 ± 0.019	-0.109 ± 0.010	+0.101 ± 0.007	+0.099 ± 0.007
	No Rnaseq, Rppa, Wsi	-0.250 ± 0.049	+0.143 ± 0.053	+0.167 ± 0.044	-0.172 ± 0.022	+0.098 ± 0.027	+0.110 ± 0.023
**256**	No Cna	-0.022 ± 0.014	+0.017 ± 0.014	+0.017 ± 0.014	-0.028 ± 0.007	+0.030 ± 0.007	+0.029 ± 0.007
	No Rnaseq	-0.025 ± 0.017	+0.026 ± 0.016	+0.023 ± 0.016	-0.178 ± 0.016	+0.177 ± 0.016	+0.177 ± 0.016
	No Rppa	-0.037 ± 0.015	+0.035 ± 0.013	+0.035 ± 0.010	-0.021 ± 0.006	+0.026 ± 0.007	+0.026 ± 0.006
	No Wsi	-0.041 ± 0.015	+0.036 ± 0.014	+0.042 ± 0.014	-0.056 ± 0.007	+0.051 ± 0.005	+0.049 ± 0.006
	No Cna, Rnaseq	-0.059 ± 0.021	+0.049 ± 0.018	+0.055 ± 0.017	-0.210 ± 0.021	+0.208 ± 0.022	+0.209 ± 0.020
	No Cna, Rppa	-0.063 ± 0.012	+0.056 ± 0.013	+0.061 ± 0.016	-0.049 ± 0.011	+0.052 ± 0.010	+0.054 ± 0.011
	No Cna, Wsi	-0.057 ± 0.026	+0.048 ± 0.018	+0.059 ± 0.019	-0.088 ± 0.010	+0.087 ± 0.010	+0.083 ± 0.011
	No Rnaseq, Rppa	-0.059 ± 0.021	+0.046 ± 0.014	+0.050 ± 0.018	-0.156 ± 0.015	+0.150 ± 0.016	+0.152 ± 0.016
	No Rnaseq, Wsi	-0.285 ± 0.027	+0.257 ± 0.022	+0.286 ± 0.029	-0.168 ± 0.013	+0.152 ± 0.013	+0.161 ± 0.014
	No Rppa, Wsi	-0.058 ± 0.014	+0.051 ± 0.007	+0.053 ± 0.012	-0.086 ± 0.013	+0.083 ± 0.012	+0.083 ± 0.011
	No Cna, Rnaseq, Rppa	-0.107 ± 0.017	+0.083 ± 0.015	+0.091 ± 0.014	-0.210 ± 0.020	+0.197 ± 0.017	+0.202 ± 0.020
	No Cna, Rnaseq, Wsi	-0.329 ± 0.051	+0.286 ± 0.048	+0.327 ± 0.046	-0.199 ± 0.014	+0.175 ± 0.016	+0.192 ± 0.016
	No Cna, Rppa, Wsi	-0.110 ± 0.029	+0.102 ± 0.023	+0.107 ± 0.019	-0.106 ± 0.015	+0.106 ± 0.013	+0.105 ± 0.014
	No Rnaseq, Rppa, Wsi	-0.302 ± 0.042	+0.178 ± 0.038	+0.202 ± 0.042	-0.165 ± 0.019	+0.089 ± 0.026	+0.111 ± 0.018

Table B. Quantitative Impact of Generative Completion across Latent Dimensions. The table compares the performance recovery for tumor stage classification and survival analysis for embedding dimensions 64, 128, and 256. Values are reported as mean ± standard deviation across 10 experimental runs.

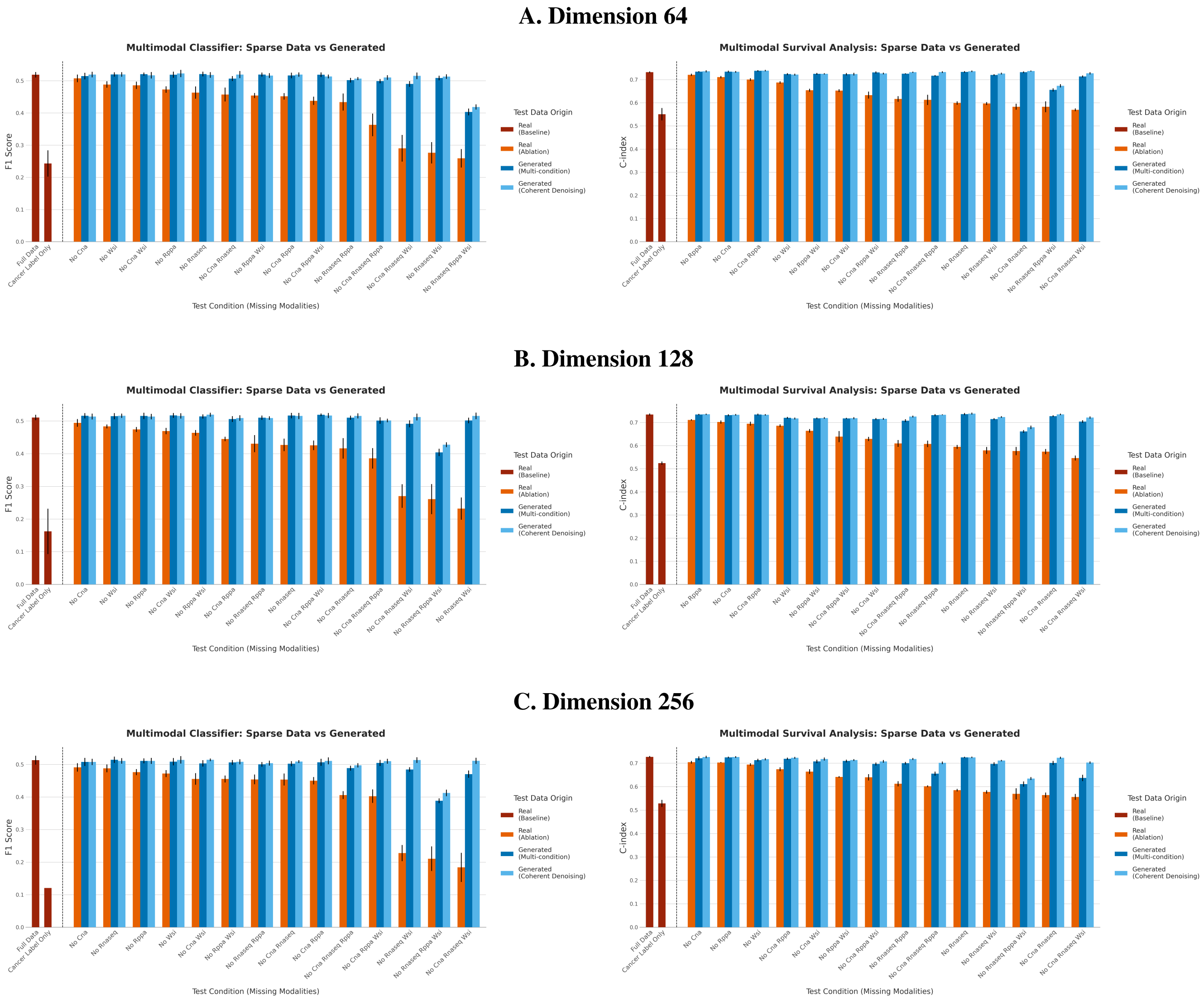

Figure B. Downstream Performance Recovery at Higher Dimensions. Performance comparison for tumor stage classification (Left column) and survival analysis (Right column) using embedding dimensions 64 (A), 128 (B), and 256 (C). The plots compare the Baseline (complete data) against Ablation (missing data) and the two generative imputation strategies (Multi-condition and Coherent Denoising). Error bars represent standard deviation across 10 experimental runs. The results indicate that the framework's ability to recover predictive signals remains robust and effective even as the dimensionality of the latent space increases.

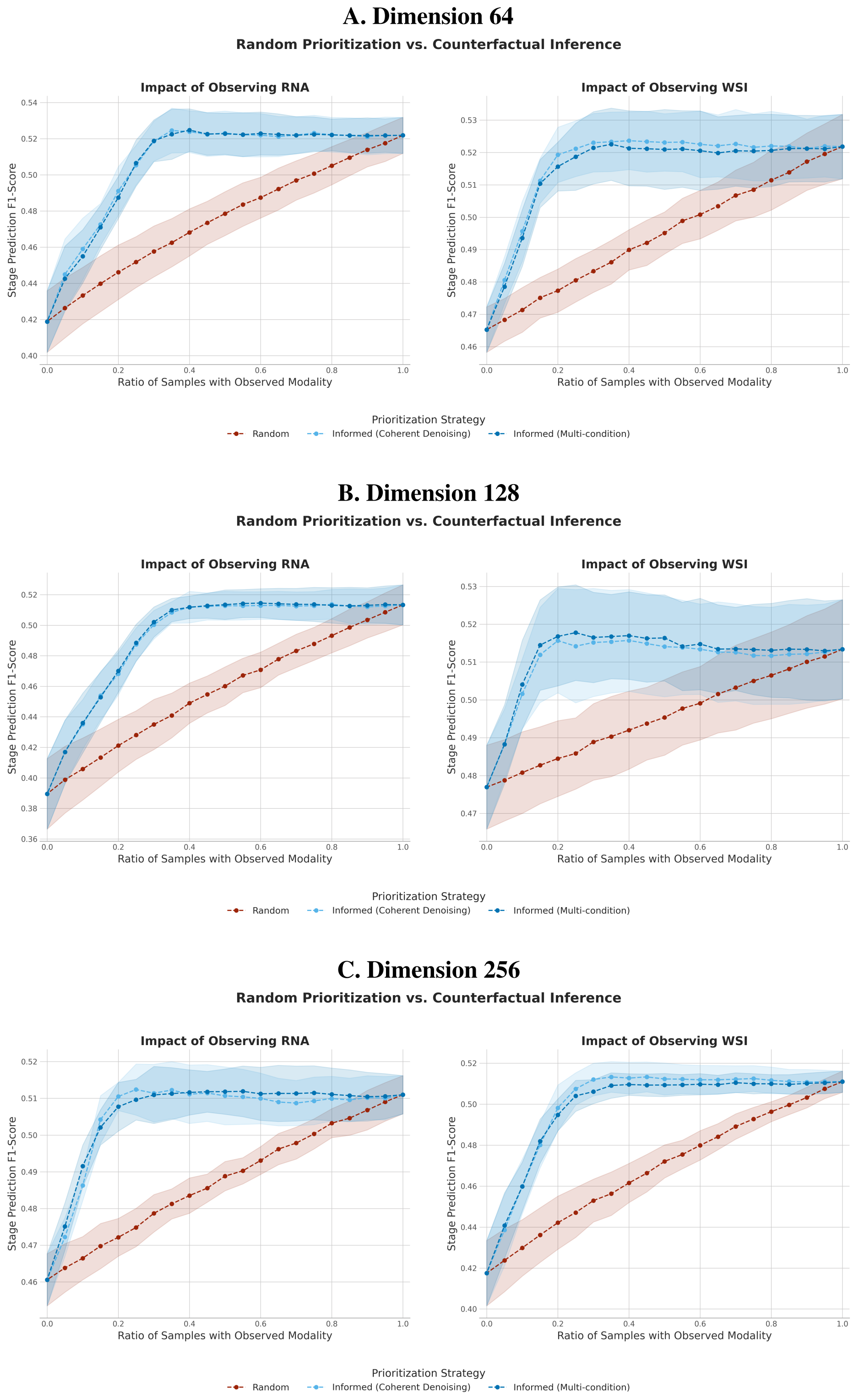

Figure C. Robustness of Counterfactual Prioritization across Latent Dimensions. Evaluation of the Informed Prioritization strategy for acquiring RNA-Seq (Left plots) and WSI (Right plots) data across embedding dimensions 64 (A), 128 (B), and 256 (C). The curves compare the standard Random Prioritization (red) against the model-guided Informed Prioritization (blue), which uses counterfactual variance to select the most informative patients first. Error bands indicate standard deviation across 10 repetitions. The persistent gap between the blue and red curves across all dimensions confirms that the generative model successfully captures patient-specific utility even in higher-dimensional latent spaces.
